# Ruxolitinib for the treatment of acute and chronic graft-versus-host disease in children: a systematic review and individual patient data meta-analysis

**DOI:** 10.1038/s41409-024-02252-z

**Published:** 2024-02-24

**Authors:** Francesco Baccelli, Francesca Gottardi, Edoardo Muratore, Davide Leardini, Antonio Giacomo Grasso, Davide Gori, Tamara Belotti, Arcangelo Prete, Riccardo Masetti

**Affiliations:** 1 Pediatric Hematology and Oncology, IRCCS Azienda Ospedaliero-Universitaria di Bologna, Bologna, Italy; 2 Department of Medical and Surgical Sciences (DIMEC), University of Bologna, Bologna, Italy; 3 Department of Biomedical and Neuromotor Sciences (DIBINEM), University of Bologna, Bologna, Italy

**Keywords:** Drug development, Haematological cancer

## Abstract

Steroid-refractory graft-versus-host disease (SR-GvHD) represents a major complication of pediatric allogenic hematopoietic stem cell transplantation. Ruxolitinib, a selective JAK 1–2 inhibitor, showed promising results in the treatment of SR-GvHD in adult trial, including patients >12 years old. This systematic review aims to evaluate ruxolitinib use for SR-GvHD in the pediatric population. Among the 12 studies included, ruxolitinib administration presented slight differences. Overall response rate (ORR) ranged from 45% to 100% in both acute and chronic GvHD. Complete response rates (CR) varied from 9% to 67% and from 0% to 28% in aGvHD and cGvHD, respectively. Individual-patient meta-analysis from 108 children under 12 years showed an ORR and CR for aGvHD of 74% and 56%, respectively, while in cGvHD ORR was 78% but with only 11% achieving CR. Treatment-related toxicities were observed in 20% of patients, including cytopenia, liver toxicity, and infections. Age, weight, graft source, previous lines of therapy, and dose did not significantly predict response, while a higher rate of toxicities was observed in aGvHD patients. In conclusion, ruxolitinib shows promising results in the treatment of SR-GvHD in children, including those under 12 years. Specific pediatric perspective trials are currently ongoing to definitely assess its efficacy and safety.

## Introduction

Despite improvement in preventive strategies, graft-versus-host disease (GvHD) represents a significant cause of morbidity and mortality in pediatric patients undergoing allogenic hematopoietic stem cell transplantation (HCT) [1, 2]. Acute GVHD (aGvHD) affects up to 50% of children undergoing HCT, while 20% present a grade II-IV aGVHD [3]. The incidence of chronic GVHD (cGvHD) is lower, between 6 and 33%, with higher risk after peripheral blood stem cell (PBSC) HCT and in patients with previous aGvHD [4, 5]. Systemic corticosteroids represent the standard first-line therapy for both acute and chronic GvHD but only half of patients respond to steroids [4–7]. The agreement on definition of steroid-refractory (SR) aGvHD has been reached in recent years, with the aim of ensuring standardized diagnostic criteria for participants in clinical trials. SR aGvHD is defined as one of the following: disease progression after 3 days of treatment with methylprednisolone (MP) 2 mg/kg per day equivalent; lack of improvement after 7 days of treatment with MP 2 mg/kg per day equivalent; progression to a new organ after treatment with MP 1 mg/kg per day equivalent for skin and upper gastrointestinal (GI) GvHD; or disease recurrence during or after a corticosteroid taper [8]. cGvHD is defined SR when manifestations progress despite the use of a regimen containing prednisone at ≥1 mg/kg per day for at least 1 week or persist without improvement despite continued treatment with prednisone at ≥0.5 mg/kg per day or 1 mg/kg every other day for at least 4 weeks. Steroid-dependent cGvHD may be defined when prednisone doses >0.25 mg/kg per day or >0.5 mg/kg every other day are needed to prevent recurrence or progression of manifestations as demonstrated by unsuccessful attempts to taper the dose to lower levels on at least 2 occasions, separated by at least 8 weeks [9]. Several second-line treatments have been proposed for SR GvHD in both acute and chronic settings, including anti–TNF-α antibodies, mycophenolate mofetil, methotrexate, anti–IL-2R antibodies, extracorporeal photo apheresis (ECP) [2, 10–12]. All these immunosuppressive drugs showed suboptimal results and are associated with high rate of complications due to profound immunosuppression [13, 14]. Pediatric experience is mainly derived from adult studies. No consensus exists regarding treatment of SR GvHD and the prognosis for patients with SR GvHD still remains poor with long term survival of 5–30% [1, 15]. These considerations lead to an urgent need for targeted therapies with higher response rate and minimal immunosuppression level. Ruxolitinb is an oral selective Janus kinase (JAK) 1 and 2 inhibitor, firstly approved for the treatment of myelofibrosis [16]. JAK 1/2 are intracellular kinases that cause the activation of signal transducer and activation (STAT) proteins, involved in proliferation, activation and survival of cells [16]. The role of this pathway is critical in T lymphocyte function, involved in activation, survival and lineage commitment [17]. It is also important in innate immune response [16]. The pathway has thus been studied with increasing interest as a potential target in immune disorders [18–20]. JAK-STAT signaling demonstrated a major role in GvHD pathogenesis [21]. Ruxolitinb was tested in preclinical models in which JAK-STAT blockade demonstrated to control clinical features of GvHD [22]. Notably, ruxolitinib demonstrated to preserve graft versus leukemia (GvL) effect in in vitro models [23]. Following these reports, ruxolitinib was firstly evaluated in acute SR GvHD showing promising results in retrospective adult studies [22, 24]. The impact of ruxolitinib on cGvHD was also reported in retrospective studies with high response rate [24–27]. The mentioned encouraging reports led to the prospective trial REACH1 (NCT02953678), an open-label, single-arm, multicenter trial of ruxolitinib in patients 12 years and older with SR and steroid-dependent aGvHD showing an overall response rate at any time of 73% with complete response rate of 56% [8]. Results of this trial led to the approval of ruxolitinib for treatment of SR GvHD by the FDA in 2019 and by EMA in 2021 [28]. Two large multicenter, randomized, open-label, phase 3 trials, REACH2 (NCT02913261) and REACH3 (NCT03774082), demonstrated the efficacy of ruxolitinib in SR aGvHD and cGvHD, respectively, with greater overall response (62% vs 39% for 28 days aGvHD response, and 49% vs 25% for week 24 cGvHD response) and greater failure-free survival in the ruxolitinib group compared to control best available therapies. Pediatric patients with 12 to 18 years of age were included in the two analysis [29, 30]. Pediatric experiences of ruxolitinib in GvHD have been increasingly reported worldwide in recent years, also including patients <12 years of age, that were excluded in the randomized clinical trials. In this specific cohort, the use of ruxolitinib in SR GvHD is still off-label. Peculiar concerns include pediatric dosing, prospectively evaluated in REACH trials between 12–18 years, but still lacking in <12 years children, as well as toxicities [31, 32]. Moreover, a difference between pediatric and adult settings is that younger children present a lower incidence of cGvHD, also related to the limited use of PBSC. Furthermore, a consistent percentage of patients receives transplantation for a non-malignant disease, making the control of transplant related mortality and prevention of severe GvHD a particularly relevant issue in pediatric HCT recipients [2, 12]. The aim of this review is to provide a systematic review on current evidence about the use of ruxolitinib for SR GvHD in the pediatric population. The two parts of the analysis include a first “qualitative” analysis on ruxolitinib administration, response, and toxicities in pediatric SR GvHD and a second “quantitative” analysis on available single-patient data about children <12 years. The clinical relevance of this review is particularly significant for this class of age, considering the lack of solid evidence in children < 12 years that will be obtained by the ongoing perspective pediatric trial REACH4.

## Methods

### Literature search

This systematic review was conducted according to the Preferred Reporting Items for Systematic Reviews and Meta-Analyses (PRISMA) guidelines [33]. The systematic review was registered on PROSPERO (ID CRD42022371905). Electronic databases, namely, PubMed and Trip, were searched to identify relevant studies up to May 2023. The following string was used to perform the literature search: (ruxolitinib OR JAK inhibitor) AND (graft versus host disease OR graft-versus-host disease OR GvHD OR aGvHD OR cGvHD). The search was restricted to English-language studies that involved pediatric allo-HCT recipients <18 years also including patients under 12 years of age, analyzing the use of ruxolitinib for the treatment of steroid refractory GvHD, both acute and chronic.

The types of studies considered eligible for this systematic review were randomized clinical trials and observational studies, both retrospective and prospective. Case reports and other systematic reviews or meta-analyses were excluded. Two reviewers (F.B. and F.G.) independently identified potentially eligible studies by screening titles and abstracts. The same authors assessed the full texts of potentially relevant studies for inclusion and consulted the references of previously published primary and secondary papers to manually search for additional relevant papers. Any disagreement regarding eligibility and inclusion in the systematic review was resolved through discussion and consensus between the 2 readers. If consensus was not reached, the opinion of a third author (E.M.) who acted as a “blind” final arbiter was requested. Investigators and corresponding authors were contacted to obtain additional information about studies with incomplete data regarding patients under 12 years of age.

### Data extraction

We used the same methodology for data extraction, performed independently by the same 2 reviewers (F.B. and F.G.) under the supervision of a third author (E.M.). Data were summed and analyzed using Microsoft Office Excel 2013 (Microsoft, Redmond, WA), GraphPad Prism version 8.0.0 for Windows (GraphPad Software, San Diego, California USA) and IBM SPSS Statistics for Windows, 22.0 (IBM Corp., Armonk, NY, USA)). We extracted data regarding ruxolitinib administration, response rate and toxicities both in the setting of acute and chronic GvHD.

### Quality assessment

Quality assessment was performed independently by two authors (F.B. and E.M.) and any disagreement was resolved through discussion and consensus between the two authors. We used the Strengthening the Reporting of Observational Studies in Epidemiology (STROBE) statement to assess the quality of the observational studies included in the meta-analysis. The STROBE statement is a 22-item tool specifically designed to evaluate the quality of cohort studies [34]. Items are associated with different sections of an article, such as title and abstract (item 1), introduction (items 2 and 3), methods (items 4–12), results (items 13–17), discussion (items 18–21), and other information (item 22 for funding). Eighteen items are identical for 3 different study designs, whereas 4 items (items 6, 12, 14, and 15) are differentially intended for a specific study type (ie, cohort or case-control study). The STROBE statement does not provide scoring stratification. As a rule, the higher the score, the higher the quality of the study. Thus, we created 3 score thresholds corresponding to 3 levels of quality: 0 to 14 was considered low quality; 15 to 25, intermediate quality; 26 to 33, high quality.

### Analysis

We performed two types of analysis: firstly, we pooled together data regarding ruxolitinib administration, response, and toxicities both in the setting of aGvHD and cGvHD for all pediatric cohorts (<18 years) including also patients under 12 years of age in the so-called “qualitative” synthesis. Then, we analyzed selectively available single-patient data about children <12 years regarding response rate to ruxolitinib (“quantitative” synthesis).

The following outcomes were evaluated in descriptive analysis, when available: overall response rate (ORR), complete response (CR), partial response (PR), non-response (NR), treatment failure (TF), time to achieve response, overall survival (OS) and treatment-related toxicity (TRT). CR was defined as complete resolution of GvHD symptoms, PR as improvement in the stage of GvHD without worsening in other organ, NR as no improvement or deterioration of GvHD symptoms or development of GvHD symptoms in other organs, TF as discontinuation of ruxolitinib due to toxicities. Data about single patients <12 were collected in a database to calculate the previous mentioned outcomes of this cohort in the “quantitative” analysis. In this sub-analysis, frequencies were estimated after pooling data from different contributing authors, and then reported in subgroups of interest based on GvHD characteristics (aGvHD, cGvHD, gut aGvHD, chronic lung GvHD), daily dose received, ( ≤ and >10 mg), and previous treatments (1–2 vs >2 lines of therapy). A backward stepwise linear regression was used to identify possible predictors of the outcomes, namely ORR and TRT, out of candidate variables age, weight, source of stem cell, acute GvHD (vs chronic), previous lines of therapy, and dose. At each step, variables were chosen based on *p*-values, and the p-value threshold of 0.2 was used to set a limit on the total number of variables included in the final model.

## Results

### Literature search

**Fig. 1:**
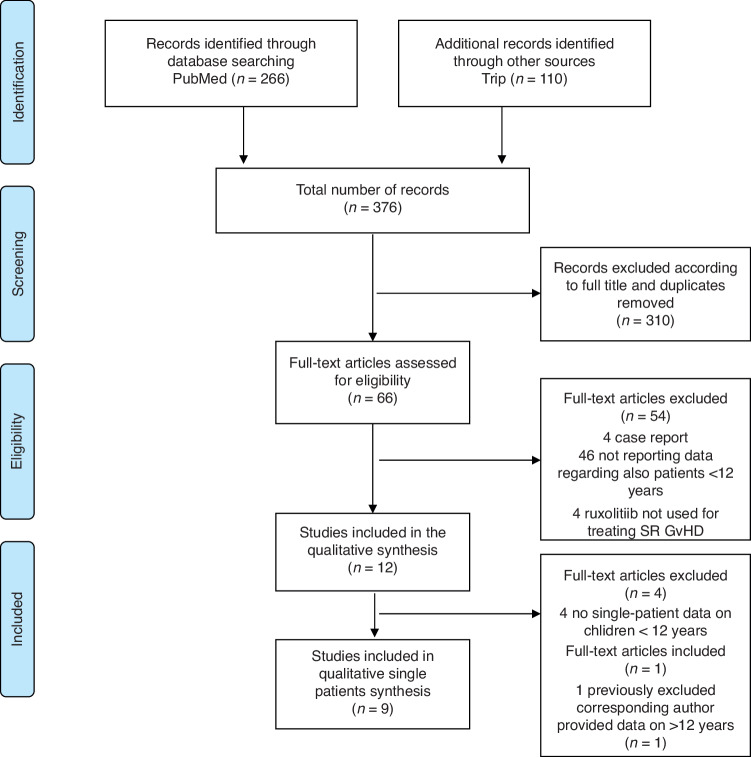
PRISMA flow diagram of the search strategy and included studies. The relevant number of papers at each point is given.

The literature search strategy identified a total of 376 references (266 in PubMed, 110 in Trip). The number of potentially relevant record identified by full titles was 66. Among these papers assessed of eligibility, 4 were excluded from the systematic review because they were case reports, 4 because they addressed the use of ruxolitinib for a different indication than from the treatment of SR GvHD, and 46 did not report data of pediatric patients including also those <12 years (Fig. 1). Of the 12 included studies in the qualitative synthesis, all but one [35] were retrospective cohorts. Two of the retrospective studies analyzed multicentric cohorts [36, 37]. 7 studies included both aGvHD and cGvHD patients, 3 studies evaluated only aGvHD patients and 2 only cGvHD. In 7 studies, only pediatric patients were included [37–43] whereas the other 5 studies included also adult patients, but outcomes were reported separately. The studies by Moiseev et al. [35] and Wei et al. [44] do not report specific data about pediatric patients but were included in the qualitative synthesis because authors provided a statistical analysis founding no differences in results between adult and pediatric patients. Four studies report single-patient data for children <12 years while, for 5 other studies, corresponding authors provide these details. The paper by Escamilla Gomez et al. [36], a retrospective, multicentric study on both adult and pediatric patients, was firstly excluded from the qualitative synthesis because it did not report data regarding pediatric patients <18 years separately but was included in the single-patient analysis because the corresponding author provided data about patients <12 years. In four of the 12 studies, single-patient data regarding response rate to Ruxolitinib in children <12 years were neither available neither provided by corresponding authors, and were therefore excluded from the quantitative synthesis. In conclusion, quantitative synthesis was performed in 9 studies for a total of 108 patients [36, 38, 40–42, 44–47] The quality of the included studies was assessed as described in Methods and reported in Table 1.

**Table 1:** Summary of included studies.

Author, year	Study design, years	aGvHD (n. of pediatric patients)	cGvHD (n. of pediatric patients)	Pediatric only vs mixed population	N. of patients < 12 years of age	Included in < 18 years analysis	Included in single-patient < 12 years analysis^a^	Quality assessment^e^
Khandelwal, 2017	Retrospective, monocentric, 2013–2016	Acute (11)	/	Pediatric only	8	Yes	Yes	high
Gonzalez Vicent, 2018	Retrospective, monocentric, 2016–2018	Acute (13)	chronic (9)	Pediatric only	11 + 8	Yes^g^	No	intermediate
Schoettler, 2019	Retrospective, monocentric, 2010–2018	/	Chronic [BOS] (4)	Pediatric and adult	3	Yes	Yes	intermediate
Uygun, 2020	Retrospective, monocentric, 2014–2018	Acute (13)	Chronic (16)	Pediatric only	10 + 9	Yes	Yes^f^	intermediate
Laisne, 2020	Retrospective, multicentric (15 centers, France), 2014–2017	Acute (29)	/	Pediatric only	25	Yes	Yes^f^	intermediate
Meng, 2020	Retrospective, monocentric, 2017–2019	Acute (3)	/	Pediatric and adult	1	Yes	Yes	intermediate
Moiseev, 2020	Prospective, single-center open-label study (NCT0 2997280); 2016–2018	Acute (17)	Chronic (17)	Pediatric and adult	N/A	Yes^d^	No	high
Escamilla Gomez, 2020	Retrospective, multicentric (13 centers, Spain), 2015–2017	Acute (N/A)	chronic (N/A)	Pediatric and adult	5 + 6^b^	No	Yes^f^	intermediate
Mozo, 2021	Retrospective, multicentric (2 centers, Spain), 2017–2018	Acute (8)	Chronic (12)	Pediatric only	N/A	Yes	No	intermediate
Yang, 2021	Retrospective, monocentric, 2017–2019	Acute (17)	Chronic (36)	Pediatric only	30^c^	Yes	Yes^f^	low
Wei, 2021	Retrospective, monocentric, 2017–2020	Acute (4)	Chronic (1)	Pediatric and adult	1 + 1	Yes^d^	Yes^f^	intermediate
Marcuzzi, 2022	Retrospective, monocentric, N/A	Acute (6)	Chronic (6)	Pediatric only	N/A	Yes	No	low
Wang, 2022	Retrospective, monocentric N/A	/	Chronic (16)	Pediatric and adult	7	Yes	Yes	intermediate

*aGvHD* acute graft versus host disease, *BOS* bronchiolitis obliterans, *cGvHD* chronic graft versus host disease, *N/A* not available, *n.* number.

^a^only studies with available data regarding single patients <12 years were included in this analysis. Investigators and corresponding authors were contacted in order to obtain this information.

^b^data regarding pediatric patients have not been provided in the paper but corresponding author provided specific data about <12 years patients and thus they were included in the single patient analysis <12 years.

^c^specific data for aGvHD versus cGvHD were not available for <12 years patients of this study.

^d^no specific data were available for pediatric patients included but authors provided a statistical analysis the revealed no significant difference between pediatric and adult patients in the two studies.

^e^Quality assessed using the Strengthening the Reporting of Observational Studies in Epidemiology (STROBE) for observational cohorts.

^f^Single patient <12 years data provided by the corresponding authors.

^g^number of patients <12 years was assumed by Ruxolitinib doses administered but single patient data were not available for this paper.

### Descriptive analysis

#### Ruxolitinib administration

Ruxolitinib administration presented slight differences among the reported cohorts. Details are reported in Supplementary Table S1. Regarding initial dose, most of the studies started with a dose of 2.5 or 5 mg twice daily according to patient weight (more or less than 25 kg) in children <12 years. In patients >12 years a dose of 10 mg twice daily was generally adopted, analogous to adult dose in REACH trials [29, 30]. In the French cohort a median dose according to body surface was reported (12.6 and 12.8 mg/m2/day in <6 and >6 years old patients) [41]. In the prospective study by Moiseev et al, the initial dose in patients <40 kg was 0.15 mg/kg/day while patients weighting >40 kg were treated with adult dose [35]. Dose reduction was performed for patients treated with azoles or with chronic renal insufficiency in some included studies [37, 41, 45, 47]. In the majority of studies Ruxolitinib was escalated to the maximum adult dose of 10 mg twice daily in the absence of toxicities. Dose reduction was performed in the studies with different intents, specifically for maintenance [42, 43] or for adverse events [46]. Time to start of treatment after diagnosis of GvHD varies among different studies, from a minimum value of 5 days in aGvHD and a maximum of 18 months in cGvHD [35, 39]. Ruxolitinib administration was almost invariably preceded by other lines of immunosuppressant therapies ranging from 1 to 6 lines. Median continuation of ruxolitinib treatment reported ranges from 14 days to 1127 days [37, 47]. Ruxolitinib was administered both in monotherapy and in combination with other immunosuppressant drugs such as calcineurin inhibitors, sirolimus, mycophenolate mofetil, ECP, anti-TNF antibodies, and rituximab [35].

#### Response in aGvHD

In eight of the 12 studies included in the qualitative analysis, data about children with aGvHD treated with ruxolitinib were available [37–43, 46]. In two other studies that comprised adult and pediatric patients, pediatric specific data were not available but there was no significant difference in response rate when stratified by age, and were thus included in the qualitative synthesis [35, 44]. In the total 10 studies, ORR to Ruxolitinib at any time varies from 45% to 100%, with complete response (CR) of 9% to 67.5%. TF was reported in a range of 17–36% and absence of response varies from 0% to 25%. Median time to response, including PR, was variable from 5 to 68 days, when the information was available [35, 41–44]. In NCT02997280 prospective study by Moiseev et al., patients with grade III–IV GvHD had significantly reduced ORR. Furthermore, liver GvHD severity and grade IV GI GvHD were associated with worse response rate, and a trend with longer time to response in patients with GI involvement was reported. Nevertheless, none of the transplant and donor characteristics were predictive for response in acute GvHD patients. [35]. In the retrospective study by Laisne et al, including 29 children, no association of baseline characteristics and CR/PR to ruxolitinib was found. Neither the number of immunosuppressive agents before using ruxolitinib nor involved organs was significantly associated with response [41]. Specific data regarding response rate according to organ involved (skin, liver or gut) and grade III-IV, in the 10 included articles are detailed in Table 2.

**Table 2:** Response in pediatric patients with aGvHD among the included papers.

Author	N. of pts	S (%)	OR (%)	L(%)	OR(%)	G(%)	OR (%)	III-IV (%)	OR in III-IV (%)	ORR %	CR %	NR %	TF %	TTR days	TTCR days
Khandelwal	11	7 (53)	2 (28)	2 (15)	0	12 (92)	3 (25)	11 (84)	NA	45	9	18	36	NA	NA
González Vicent	13	8 (61)	(75ORR 33 CR)	4 (31)	(73ORR18 CR)	6(46)	77 ORR31 CR	13 (100)	(77)	77	31	23	NA	NA	NA
Uygun	13	NA	NA	NA	NA	11 (85)	(82)	11 (85)	(82)	85	69	15	NA	NA	NA
Laisne	29	18 (62)	5/6 with isolated skin (83)	4 (14)	NA	20 (69)	15/20 (75)	22 (76)	16/22 (72)	72.4	65.5	NA	27.6	69	41
Meng	3	6 (50)	(100)	9 (75)	(78)	8 (67)	(87)	6 (50)	5/6 (83)	83.3	58.3	0	17	NA	NA
Moiseev	17	NA	NA	NA	NA	NA	NA	NA	NA	75	63	25	NA	20	53
Mozo	8	1 (12)	(100)	0	NA	7 (87)	6/7 (86)	(100)	(87.5)	87.5	37	12	NA	28	NA
Yang	17	NA	NA	NA	NA	NA	NA	13 (76)	NA	64.7	29.4	0	35	28 (to best resp)	NA
Wei	4	NA	NA	NA	NA	NA	NA	NA	NA	86.9	56.5	13	NA	5	NA
Marcuzzi	6	NA	NA	NA	NA	NA	NA	2 (33)	(100)	100	100	NA	NA	21	NA

*CR* complete remission, *G* gut, *L* liver, *N.* number, *NA* not applicable, *NR* non response, *OR* overall response, *ORR* overall response rate, *S* skin, *TF* treatment failure, *TTCR* time to complete response TTR, time to response.

#### Response in cGvHD

Nine studies described treatment with ruxolitinib in children with SR cGvHD. Of them, while seven reported specific response data in children [37, 39, 40, 42, 43, 45, 47], two did not provide specific data by age but no significant difference in response was observed when stratified by age, and were thus considered [35, 44]. Overall response rate (ORR) was variable from 50% to 100%, with complete response (CR) from 0 to 28%. Median time to response was variable from 21 to 71 days [35, 37, 42, 45]. In the prospective study NCT02997280, none of the transplantation and donor characteristics were predictive for response. The analysis of final severity revealed that there was a significant reduction in the scores of skin severity, mouth mucosa, GI tract, and liver. On the other hand, the changes in the severity scores of eyes, lungs, joints, and genitalia were not significant [35]. Ruxolitinib performance on lung GvHD/bronchiolitis obliterans (BOS) was evaluated in five studies with variable but generally high response rate (50–90%) [37, 39, 40, 45, 47]. Data regarding studies investigating Ruxolitinib treatment responses in cGvHD are summarized in Table 3.

**Table 3:** Response in pediatric patients with cGvHD among the included papers.

Author	N. of pts	Sclerodermic features n.	OR skin %	Lung involvement n.	OR lung %	ORR %	CR %	PR %	NR %	TTR days
Gonzalez Vincent	9	6	100	7	86	89	22	67	11	NA
Schoettler	4	2	NA	4	50	50	0	50	50	21
Uygun	16	8	75	7	71	71	6.5	75	18.5	NA
Moiseev	17	NA	NA	NA	NA	81	20	61	19	71
Mozo	12	8	100	10	90	91	8	84	8	60
Yang	36	NA	NA	NA	NA	81	28	53	14	68
Wei	1	NA	NA	NA	NA	78	25	53	22	NA
Marcuzzi	7	NA	NA	NA	NA	100	0	100	0	NA
Wang	16	7	100	3	67	69	12.5	56.5	31	NA

*CR* complete remission, *N.* number, *NA* not applicable, *NR* non response, *OR* overall response, *ORR* overall response rate, *PR* partial response, *TTR* time to response.

#### Toxicities

Ruxolitinib was generally well tolerated in the ten studies reporting specific toxicity data in pediatric patients. When reported, the discontinuation rate due to toxicities, ranges from 0 to 30% [37, 38, 41, 42, 45]. Adverse events mostly included cytopenia, liver toxicities and infective complications. Cytopenia represented the most frequent complication and most frequently presented as neutropenia and thrombocytopenia with a weighted average incidence of 26% (range, 0–100) and 20% (range, 0–100), respectively. Cytopenia was managed by transfusions [37, 38], colony-stimulating factor support [38], dose reduction and discontinuation [47, 48]. Regarding thrombocytopenia, in the study by Meng and colleagues, 3 pediatric patients with grade 3–4 thrombocytopenia received thrombopoietin agonists [46]. Liver toxicity was reported frequently during the treatment and, in some cohorts, it was present in all the treated patients [37]; however, rarely it was a cause of discontinuation [47]. Infections were reported as frequent complication of ruxolitinib as well. Bacterial infections presented with variable clinical presentation, ranging from non-serious infections [39] to death-causing ones [31]. Viral infections or reactivations were common during ruxolitinib therapy, with a weighted average incidence of 30% (range, 0–75). CMV reactivation was common [35–37, 39, 40, 42, 43, 46–48]; however, no death related to CMV disease occurred. Monitoring of viral replication was performed routinely during transplant procedure in all studies, with start of pre-emptive therapy in case of CMV reactivation. No mention of anti-CMV prophylaxis was reported in any of the included studies. Other viral infections included EBV [35, 38, 39, 41, 47, 48], BK virus [35, 37, 38, 42, 47], HHV6 [39] and adenovirus [38]. One patients developed an adenovirus-sustained liver infections and deaths related to disseminated adenovirus infections [39, 41]. Fungal infections were far less frequent, mostly related to *Candida* [38] *Aspergillosis* [37, 39] *Pneumocystosis* [39, 42]. Details of adverse events reported in the included studies are summarized in Supplementary Table S2.

#### Survival

10 of the 12 studies included in the qualitative synthesis provided data regarding survival in patients treated with ruxolitinib. Six studies described the occurrence and cause of death collected through the day of the last follow-up, while in 5 studies estimated OS and/or EFS were calculated. Detailed information for each study is shown in Supplementary Table S3. When OS analysis were performed, higher survival for cGvHD patients was almost always reported for patients receiving ruxolitinib. OS ranged from 30% to 92.3%, and from 76.4% to 100% for aGvHD and cGvHD, respectively. Median time of follow up was described very heterogeneously in the included studies, starting either from HCT, diagnosis of GvHD or start of ruxolitinib treatment. Specific data are reported in the supplementary table. In two studies, parameters associated with survival were addressed. Interestingly the rate of ORR in the aGvHD setting was significantly associated with survival [41]. In the work by Moiseev and colleagues the worse outcome in aGvHD group remained significant even when corrected for underlying disease risk and response to ruxolitinib, although the analysis regarding adults and children were not reported separately. However, no difference in OS was observed between adults and children (65% vs 53%, *p* = 0.44) considering both acute and chronic GvHD patients. The major factors predicting survival were grade III–IV GI involvement and the underlying disease risk in the aGvHD and cGvHD, respectively [35].

### Single-patient analysis of children <12 years

#### Patients

Single-patient data were available for 108 patients from 9 studies. Main patient, disease and transplant characteristics are reported in Table 4. The median age was 6 years (range 1–12). Seventy-two (67%) patients received HCT for non-malignant and 36 (33%) for malignant diseases. Fifty-one (65%) patients were treated for aGvHD and 27 (35%) for cGvHD, while 29 patients were not reported as treated for acute or chronic. All 108 patients were previously treated with steroids and received a median of 3 further lines of immune-suppressive therapies before ruxolitinib (range 1–7). More frequently used second-line therapies before ruxolitinib start were TNF inhibitors, calcineurin inhibitors (CNI), extracorporeal photopheresis (ECP), sirolimus and mesenchymal stromal cells (MSC).

**Table 4:** Baseline characteristics of patients <12 years.

Patients, number (%)	108
Age, median (range)	6 years (1–12)
• ≤6 years	• 52 (48)
• >6 years	• 56 (72)
Disease, number (%)	
• Malignant	72 (67)
• Non-malignant	36 (33)
Conditioning, number (%)	NA in 30
• Myeloablative	30 (38)
• Reduced intensity	39 (50)
• Non myeloablative	9 (12)
Donor, number (%)	NA in 30
• Matched related donor	12 (15)
• Matched unrelated donor	50 (65)
• Mismatched ( ≤ 7/8) unrelated donor	10 (12)
• Haploidentical donor	6 (8)
Source, number (%)	NA in 36
• BM	49 (68)
• PBSC	16 (22)
• CB	7 (10)
Type of GvHD, number (%)	NA in 30
Acute	51 (65)
○ III-IV	○ 29 (57)
○ Gut	○ 39 (76)
Chronic	27 (35)
○ Severe	○ 12 (44)
○ Lung	○ 12 (44)
Previous therapies, number (%)	NA in 30
• median (range)	3 (1–7)
• TNF inhibitors	27 (35)
• CNI	28 (36)
• ECP	25 (32)
• Sirolimus	24 (31)
• MSC	18 (23)
• Other (basiliximab, ibrutinib, tocilizumab)	25 (32)

*NA* not available, *BM* bone marrow, *PBSC* peripheral blood stem cells, *CB* cord blood, *TNF* tumor necrosis factor, *CNI* calcineurin inhibitor, *ECP* extracorporeal photopheresis, *MSC* mesenchymal stromal cells.

#### Ruxolitinib administration

Seventy-one of the included patients had a weight <25 Kg and twenty-two >25 kg. Overall, median starting daily dose was 10 mg (range 2.5–20). Twenty-four out of 51 (47%) patients <25 kg received a starting daily dose of 5 mg (2.5 mg BID) while 19/51 (37%) of 10 mg (5 mg BID). Twenty-two ot of 27 (81%) patients >25 Kg received an initial dose of 5 mg BID, while 5/27 (19%) of 10 mg BID. In 25 patients, dose was increased during treatment. When reported, the reason for dose increase was the lack of adequate response with the current dose in the absence of severe toxicities. Median dose increase was 5 mg (range 2.5–15) and 10 patients reached a maximum dose of 10 mg BID (identical to adult dose explored in REACH 2 and REACH3). In 6 patients, daily dose has been administered every 24 h and in one case every other day, whereas in all the other cases two times/day (BID). Median length of treatment was 80 days (1–610). Details are reported in Table 5.

**Table 5:** Ruxolitinib administration in patients <12 years.

Weight, number (%)	NA in 30
<25 kg	51 (65)
>25 kg	27 (35)
Daily dose, mg/die, number (%)	NA in 30
• median (range)	10 mg (2.5–20)
• Starting dose in patients <25 kg (51 pts),	
○ 10 mg/die	19 (37)
○ 5 mg/die	24 (47)
○ 2.5 mg/die	5 (10)
○ 20 mg/die	3 (6)
• Starting dose in patients >25 kg (27 pts)	
○ 10 mg/die	22 (81)
○ 20 mg/die	5 (19)
• Maximum dose reached	
• 20 mg/die	• 16 (21)
• 15 mg/die	• 7 (9)
• 10 mg/die	• 37 (47)
• 5 mg/die	• 18 (23)
Total treatment time, days, median (range)	80 (1–610) (NA in 3 patients)

#### Response

Rates of response were counted as ORR, CR, PR, TF, NR as previously defined. ORR in the whole pooled cohort was 77%, PR 38% and CR 39%. NR was reported in 5% and TF in 19%. In 51 aGvHD patients ORR was 74% with 56% CR, 4% NR and 19% TF. In the 27 cGvHD patients ORR was 78% with only 11% of patients achieving CR. NR was reported in 11% and TF in 7% of patients. Among 40 severe scored GvHD (29 aGvHD grade 3–4 and 11 moderate-severe cGVHD), ORR was 72% and 67% in acute and chronic GvHD, respectively. Out of 39 gut aGvHD, 69% were reported as responders. Patients with association of gut and liver involvement were 6, of these 2 (33%) had CR, 1 (17%) had PR, and 3 (50%) had TF. One patient had exclusive liver involvement and achieved PR. Lung cGvHD/BOS was reported in 12 patients with response in 75%. Time to achieve the response was available for 33/108 patients, with a median time to response (including both CR and PR) of 11 days (rang, 5–101). In stepwise multivariate regression analysis, none of the analyzed factors resulted significantly predictive of response, as shown in Table 6.

**Table 6:** Response analysis in patients <12 years according to age, weight, graft source, GvHD characteristics, dose and lines of previous therapies.

Patients (*N*)	CR + PR *N* (%)	TF + NR *N* (%)	Univariate analysis		Multi-variate stepwise analysis	
			Odds Ratio for response (IC 95%)	*P*-value	Odds Ratio for response (IC 95%)	*P*-value
Age						
>6 years (56)	41 (73)	15 (27)	Ref	0.37	0.29 (0.06–1.45)	0.13
<6 years (52)	42 (80)	10 (19)	0.65 (0.26–1.60)			
Weight						
>25 Kg (22)	14 (64)	8 (36)	Ref	0.16	0.84 (0.18–4.10)	0.84
<25 Kg (71)	56 (79)	15 (21)	0.47 (0.17–1.36)			
Type of GvHD					excluded by stepwise selection	-
aGvHD (51)	38 (74)	13 (25)	Ref			
cGvHD (27)	21 (78)	6 (22)	0.83 (0.26–2.4)	0.74		
Acute GvHD					-	-
III/IV aGvHD (29)	21 (72)	8 (28)	Ref			
II aGvHD (18)	14 (78)	4 (22)	0.75 (0.17–2.88)	0.68		
Gut aGvHD (39)	27 (69)	12 (31)				
Chronic GvHD					-	-
Severe cGvHD (12)	8 (67)	4 (33)	Ref	0.60		
Moderate cGvHD (6)	5 (83)	1 (17)	0.50 (0.02–5.03)			
Lung cGvHD (12)	9 (75)	3 (25)				
Source of HSC						
BM (49)	34 (70)	15 (30)	Ref	0.06	0.11 (0.01–1.02)	0.053
PB (16)	14 (87)	2 (13)	0.26 (0.04–1.05)			
CB (7)	6 (85)	1 (15)				
Previous lines of therapy						
1–2 (31)	21 (68)	10 (32)	Ref.	0.80	2.8 (0.75–10.66	0.12
>2 (47)	39 (83)	8 (17)	1,38 (0.97–3.96)			
Daily dose						
<10 mg daily (61)	45 (74)	16 (26)	Ref	0.80	1.66 (0.51–13.75)	0.25
>10 mg daily (17)	14 (82)	3 (18)	1.66 (0.47–7.87)			

*ORR* overall response rate, *CR* complete response, *PR* partial response, *NR* non-response, *TF* treatment failure, *NA* not available data about type of, *GvHD, ref* reference for categoric variables.

#### Toxicities

Data about treatment-related toxicity (TRT) were available in 80/108 (74%) patients and are reported in Supplementary Table S4. Twenty-percent presented cytopenia and the same percentage had liver toxicity. Overall rate of infections was 20%. Ten-percent of patients developed CMV reactivation and 6% bacterial infections. 53% of patients aged <12 did not present any toxicities. Toxicities during aGvHD and cGvHD were reported in 73% and 42% of patients for each subgroup, respectively. Patients with aGvHD presented with cytopenia in 26% of cases, while 17% cGvHD patients had this complication. Infections during ruxolitinib affected 22% of aGvHD and 42% of cGvHD patients. Rate of CMV reactivation was 15 and 17% in the two groups. Among the 20 cases of TF, 8 were reported as related to complications of treatment (1 case neutropenia grade 4, 1 case of not specified cytopenia, 3 cases of liver toxicity grade 3–4, 1 sepsis, 1 CMV reactivation), whereas one case was related to leukemia relapse and for 11 patients this data is not available. In the stepwise multivariate regression analysis, patients treated for acute GvHD resulted affected by a significantly higher rate of toxicities than cGvHD (Odds Ratio 5.22, CI: 95%1.03–26.5, *p*-value 0.046), while no other considered parameter resulted significantly associated with toxicities rate, as reported in Table 7.

**Table 7:** Analysis of adverse events in patients <12 years according to age, weight, graft source, GvHD characteristics, dose and lines of previous therapies.

Patients (*N*)	Adverse events	No adverse events	Uni-variate analysis		Multi-variate stepwise analysis	
			Odds Ratio for adverse events (IC 95%)	*P*	Odds Ratio for adverse events (IC 95%)	*P*
Age						
>6 years (42)	21 (50)	21 (50)	Ref			
<6 years (38)	17 (45)	21 (55)	1.23 (0.51–3.00)	0.60	1.03 (0.77–1.38)	0.82
Weight						
>25 Kg (18)	11 (61)	7 (39)	Ref	0.20	2.7 (0.38–19.26)	0.32
<25 Kg (50)	22 (44)	28 (56)	2.00 (0.68–6.25)			
Type of GvHD						
Acute GvHD (26)	19 (73)	7 (27)	Ref	0.02	5.22 (1.03–26.5)	**0.046**
Chronic GvHD (24)	10 (42)	14 (58)	3.8 (1.19–13.09)			
Source of HSC					Excluded by stepwise selection	-
BM (30)	20 (67)	10 (33)	Ref			
PB (18)	8 (44)	55 (56)	1.8 (0.66–5.51)	0.24		
CB (2)	1 (50)	1 (50)				
Previous lines of therapy						
1–2 (14)	20 (56)	16 (44)	Ref	0.57	0.75 (0.45–1.26)	0.28
>2 (36)	9 (64)	5 (36)	0.69 (0.18–2.43)			
Daily dose						
>10 mg daily (11)	6 (55)	5 (45)	Ref	0.79	0.43 (0.09–28.9)	0.75
<10 mg daily (39)	23 (58)	16 (42)	0.83 (0.21–3.34)			
Starting dose					Excluded by stepwise selection	-
>10 mg daily (18)	10 (56)	8 (44)	Ref 0.86 (0.26–2.79)	0.79		
<10 mg daily (32)	19 (59)	13 (41)				

Bold values indicate statistically significant results in the multi-variate stepwise analysis.

## Discussion

Given the encouraging results of REACH2 and REACH3 trials, ruxolitinib has been increasingly administered in pediatric patients with SR GvHD, also including children <12 years [29, 30]. In this systematic review, we summarized current literature consisting of 12 papers regarding the use of ruxolitinib for SR GvHD in pediatric patients <18 years. We specifically collected single patient data about 108 younger children <12 years old that received ruxolitinib for both acute and chronic GvHD, a category of extreme interest for pediatric hematologists, given the off-label indication in this class of age [28].

Among the included studies in both the qualitative and quantitative synthesis, the initial ruxolitinib dose was generally given according to patient’s weight. Among patients <12 years, children <25 kg frequently received a dose of 5 mg daily while those >25 kg received 10 mg daily, but a certain degree of heterogeneity in the administered dose in this category of patients is present (Supplementary Table S1) and should be taken in consideration in response and toxicity analysis. Patients >12 years almost invariably received an adult dose of 10 mg BID, consistent with the dose prescribed in 12–18 years patients in the REACH2 and 3 trails [29, 30]. Dose increase was generally allowed according to toxicities and response, and 23 patients <12 years reached a dose of 20 or 15 mg/daily. Interestingly, in the French cohort study, no relationship between initial dose of ruxolitinib and response as well as time to the best response was demonstrated [41]. Similarly, in our single-patient analysis, a daily dose < or > 10 mg was not associated with response. The preliminary pharmacokinetic results of the Phase I/II REACH-4 trial (NCT03491215) on pediatric aGvHD presented at the ASH meeting in 2022 confirmed an age-appropriate recommended Phase 2 Dose (RP2D) of 5 mg BID in the 6–12 years group and of 4 mg/m2 in the <6 years group [49]. Definitive results of this trial are awaited and will certainly provide solid data in order to help pediatric clinicians, also considering the current available formulations of oral tablets, with a minimum dosage of 5 mg.

Studies in adults investigated the rate of overall response, partial response and complete response according to standardized CIBMTR criteria [50]. REACH trials considered response at day 28 as primary endpoint for aGvHD [29]. For cGvHD the primary outcome was the ORR at week 24 [30]. These time points are currently recommended as markers of efficacy in aGvHD and cGvHD [9, 51]. However, as observed by Moiseev et al., response to ruxolitinib in pediatric patients can occur later than these time points, particularly after few months in severe GI aGvHD and in cGvHD. In this view, it seems that the absence of response fulfilling PR criteria, without evidence of GVHD progression, should not be an indication to switching therapy in pediatric patinets [35].

Regarding response rates, pediatric studies seem to show results comparable to the REACH trials for both aGvHD and cGvHD, even though with high variability. The largest pediatric reports in aGvHD reported a ORR of 85%, 72.4% and 64.7% [40–42], while in cGvHD ORR of 71%, 81% and 69% [40, 42, 47] In the <12 years single patient analysis higher ORR compared to REACH trials was observed, for both aGvHD (76% vs 62.3%) and particularly cGvHD (79% vs 49.7%) [29, 30]. These positive results in younger children confirm previous findings by Laisne et al reporting that the ORR in the cohort <6 years old was higher (81.3%) than the overall pediatric population (72.4%) even if not significantly different. Very promising results were also reported from the preliminary data of the REACH4 trial showing an ORR of 84.4% at day 28 and durable ORR of 66.7% at day 56 in aGvHD. In the cGvHD setting, the limited number of patients and the lower incidence of this complication in children need to be considered. In this view, results of the pediatric trial investigating cGvHD (NCT03774082) are certainly awaited.

Patients were highly pretreated and have received a median of 3 lines of immune-suppressive therapies before ruxolitinib in the quantitative synthesis, potentially limiting the capacity to attribute the response to ruxolitinib. This potential bias has been partially overcome in the REACH trials in which patients were excluded if they have received more than 1 ore more than 2 systemic immunosuppressive therapies in addition to steroids for the treatment of SR aGvHD or cGvHD, respectively [29, 30]. Even though, number of previous therapies was not significantly associated to response in our analysis, similar to what reported by Laisne et al. [41]. More robust results will be hopefully achieved by the REACH4 study that will include, interestingly, also patient with treatment-naïve severe aGvHD, in order to explore the efficacy of ruxolitinib also as first-line therapy [49].

Regarding the time to achieve a response to therapy, this field was not completely clarified in pediatric patients and several studies do not report specific data. Moiseev et al reported a median time to PR in patients with aGvHD of 20 days to CR of 53 days, with a very wide range and a maximum time of 112 days for PR and 255 days for CR [35]. As previously mentioned, the delay to reach the best response was not influenced by dosage in another study [41]. Unfortunately, this information was available only for 33 patients <12 years in our analysis. Even thought, results are very interesting, with a median time to response (including both CR and PR) of 11 days with a range of 5–101, possibly suggesting a faster response in younger patients.

Several studies investigated different response rates according to GvHD severity and organs involved with variable results. Regarding acute GvHD, in NCT02997280 prospective study by Moiseev et al, patients with grade III–IV GvHD had significantly reduced ORR. Furthermore, liver GvHD severity and grade IV GI GvHD were associated with worse response rate, and a trend with longer time to response in patients with GI involvement was reported [35]. Reduced response in grade IV GI aGvHD was reported also in REACH2 trial, if compared to other organ involvement. Nevertheless, the highest odds ratio for response with ruxolitinib group compared to control was also reported in grade IV disease, suggesting a considerable efficacy even in this high-risk setting [29]. Involved organs were not significantly associated with response in the study by Laisne et al. [41]. In the <12 years cohort, patients with III-IV aGvHD (*n* = 29) presented a ORR of 72% while gut aGvHD (*n* = 39) reached a ORR of 69%, without a significant difference in response according to grading and gut involvement. As regards cGvHD, REACH3 founded higher ORR with ruxolitinib than with control therapy regardless of the organs involved, with odds ratios favoring ruxolitinib in all organ subgroups [30]. Interestingly, a promising good ruxolitinib performance on lung GvHD/bronchiolitis obliterans (BOS) was reported in five pediatric studies (50–90%). These data were confirmed in our <12 years analysis, with a ORR of 75% in 12 children with lung GvHD.

REACH2 and 3 showed longer failure-free survival in patients receiving ruxolitinib for both SR aGvHD and cGvHD. Authors highlighted the need for longer follow-up in order to elucidate the impact of ruxolitinib on patients’ outcomes [29, 30]. Survival data showed a certain variability in the pediatric studies reported in the qualitative synthesis, probably related to the populations analyzed, with differences in HCT indications and previous therapies received. Interestingly, Laisne et al demonstrated that response to ruxolitinib was significantly associated to higher OS [41]. A main limitation of our analysis was the lack of survival analysis in patients <12 years patients, a topic that deserves to be addressed in future pediatric trials.

Toxicities represent an important concern in the pediatric setting, particularly in younger children considering the off-label indication and the lack of authorized pediatric dosing. A good toxicity profile was generally shown, with a lower rate of severe adverse events and discontinuation rate compared to adults. Cytopenia, infections and liver toxicities appear to be the most common adverse events, similarly to adults. A discontinuation rate of 19% was reported in children <12 years, which is lower compared to the one reported in the REACH trials, for both acute and chronic GvHD [29, 30]. Even if higher rates of viral infections and cytopenia were reported when all pediatric patients were included (see Supplementary Table S2), rates of discontinuation due to toxicities, when reported, were comparable and generally lower than REACH2 and 3. These considerations suggest that younger children can present lower rates of severe cytopenia and infections justifying drug interruption. Age <6 years was not associated with a different risk of adverse events in single-patient analysis. In our analysis on patients <12 years, aGvHD was associated with the increased incidence of adverse events compared to cGvHD, confirming results of Moiseev et al. [35]. Hematological toxicity is a key issue in the early post-HCT phase due to various contributing factors, including viral reactivations and the administration of antimicrobial therapy and prophylaxis. Furthermore, patients may be cytopenic at the start of treatment due to GvHD-related immune-dysregulation.

Due to the difficulty to collect good quality and uniform information, we could not analyze the impact of immune reconstitution at the start of ruxolitinib treatment and the modulation during therapy on response rates and toxicity. This topic should be a key area of research in the near future in order to better uncover the interplay between the altered immune networks in GvHD and JAK 1/2 inhibition [35, 37, 39, 47]. Targeting both JAK1 and JAK2 with ruxolitinib has been lied to the development of cytopenia by inhibition of JAK2 signaling, considering the role of this pathway in normal hematopoiesis [52]. Interestingly, addition of selective JAK1 inhibitor itacinib versus placebo to steroids in adult patients with aGvHD was tested in a double-blinded trial (GRAVITAS-301) in order to test the efficacy of this regimen, potentially sparing normal hematopoiesis and reducing the risk of developing cytopenia. Improvement in ORR at 28 days was observed but this effect did not reach the prespecified significance level [53]. Longer term analysis of this trial, together with other studies currently ongoing on chronic GvHD (NCT04200365) will give us further information about the role of itacinib, potentially elucidating about the need of JAK2 inhibition for an effective treatment of patients with GvHD.

It has to be mentioned that, before ruxolitinib introduction, only small studies evaluated second-line therapies for aGvHD in pediatrics. Studies mainly spaced from conventional therapies (MMF, low dose MTX) to anti-cytokine drugs (anti-TNF, anti-IL2). Response rates ranged from 50 to 80% but with lower CR rates, from 46 to 56%, and high rates of infections were reported in most studies (up to 75% with anti-cytokines) [54–56]. Moreover, survival resulted less satisfactory in many studies, with OS at 1–3 years from 60 to 40% with anti-TNF therapies [57, 58]. Use of non-conventional treatments as ECP and MSC resulted in good response rates (from 66 to 80% in principal studies) but less feasibility due to technical requirements [59–61]. In this regards, ruxolitinib brings together efficacy, manageability, and lower immunosuppressive impact even in acute patients, compared to other treatment options [2, 62, 63].

In cGvHD setting low-dose MTX, imatinib and ECP were among the most commonly employed therapies in SR children before introduction of ruxolitinib. Overall responses spaced from 50 to 77% in the few pediatric studies, but, again, toxicities limited use of these strategies [55, 64, 65]. The recently introduced ibrutinib may be considered the only actual competitor in cGvHD treatment, as the IMAGINE phase I-II trial revealed 73% ORR in patients aged 1–22 years, even if low CR rates were reported in steroid refractory group (4%).] Severe adverse events rates were low, and the drug was approved by FDA for pediatric patients [66]. Nevertheless, ruxolitinib showed similar response rate in our review, with generally lower toxicity compared to aGvHD. It is important to note that pediatric cGvHD represents a multi-system disease with variable individual clinical course. In this view, a personalized approach needs to be adopted, highlighting the importance of early diagnosis and treatment and taking into consideration the clinical status and individual needs of each patient when choosing the most appropriate treatment option [62, 63, 67].

## Conclusions

The use of ruxolitinib has changed the landscape in the management of SR GvHD in adults, effectively becoming the drug of choice in the standard approach after failure of corticosteroid therapy. Promising results have also been published in pediatric patients, with the approval for children >12 years by FDA and EMA. We showed favorable results in terms of efficacy and tolerability even in children <12 years, both in acute and chronic GvHD setting, and including the high-risk categories of gut III-IV aGvHD and lung GvHD/BOS. Results of ongoing trials, particularly REACH 4, are awaited to better implement this potentially revolutionary therapy in pediatric GvHD.

### Supplementary information

supplementary

## Data Availability

The datasets generated during and/or analyzed during the current study are available from the corresponding author on reasonable request.
