# Supplementary material for: Ruxolitinib for the treatment of acute and chronic graft-versus-host disease in children: a systematic review and individual patient data meta-analysis
Source: Bone Marrow Transplant. 2024 Feb 24;59(6):765–76. doi: 10.1038/s41409-024-02252-z (PMC11161405; doi:10.1038/s41409-024-02252-z)
Supplement: Supplementary file 1 — supplementary [file 41409_2024_2252_MOESM1_ESM.docx]

**Supplementary Table S1:** Ruxolitinib administration among the included papers

| **Author, n. of patients** | **Ruxolitinib dosage** | **Median day of Ruxolitinib start** | **Prior GVHD therapies** | **Ruxolitinib duration** | **Ruxolitinib discontinuation** |
| --- | --- | --- | --- | --- | --- |
| Khandelwal, P. [15], 11 | Initial dose:  <25 kg: 2.5 mg twice daily >25 kg: 5 mg twice daily  Dose escalation:  doubled until a maximum of 10 mg twice daily | 147 days (range 55-538) after diagnosis | immune suppressive agents before ruxolitinib: 4 (range 1-6)    mPDN, basiliximab, infliximab, tocilizumab, alemtuzumab, ECP, budesonide | Continuation for median 114 (range 12-306) days | Discontinued for adverse effects (n=8) or CR (n=1) |
| González Vicent, M. [19], 22 | Initial dose:  Infants: 2.5 mg once daily  <25 kg: 2.5 mg twice daily >25 kg: 5 mg twice daily  >12 years: 10 mg twice daily | aGvHD:  9 days after diagnosis  cGvHD:  18 months after diagnosis | aGVHD:  immune suppressive agents before ruxolitinib: 3 (range 1-6)  cGVHD:  immune suppressive agents before ruxolitinib: 3 (range 2-5) | aGVHD:  Continuation for median 84 (range 16-294) days  cGVHD:  Continuation for median 452 (range 103-665) days | aGVHD:  Discontinued for CR (n=5) and for death (n=6) |
| Marcuzzi, A. [23], 12 | Initial dose:  10 mg twice daily (all patients >25 kg) | 37 days (range 31–43 days) after diagnosis | steroid, mycophenolate mofetil, infliximab, rituximab, basiliximab, ECP, fludarabine, and antithymocyte globulin | Continuation for median 122 (range 59-188) days | Discontinued for CR (n=5) |
| Wang, Y. [17], 16 | Initial dose:  <25 kg: 2.5 mg twice daily >25 kg: 5 mg twice daily  >12 years: 10 mg twice daily  Dose escalation:  increasing until a maximum of 10 mg twice daily  Dose reduction:  if azole treatment | cGvHD:  181 days (range 17-1792) after diagnosis | Not specified | Continuation for median 421 (range 42-1127) days | Discontinued for adverse events (n=2) |
| Yang, W. [20], 53 | Initial dose:  <25 kg: 2.5 mg twice daily >25 kg: 5 mg twice daily  >12 years: 10 mg twice daily  Dose escalation:  increasing until a maximum of 10 mg twice daily | Not reported | aGVHD:  immune suppressive agents before ruxolitinib: 3 (range 2-4)  cGVHD:  immune suppressive agents before ruxolitinib: 3 (range 2-6) | aGVHD:  Continuation for median 87 (range 14-305) days  cGVHD:  Continuation for median 207 (range 28-373) days | aGVHD:  Discontinued for adverse events (n=6)  cGVHD:  Discontinued for treatment failure (n=5) |
| Mozo, Y. [12], 30 | Initial dose:  <25 kg: 2.5 mg twice daily >25 kg: 5 mg twice daily  Dose escalation:  increasing until a maximum of 5 mg twice daily <12 years or 10 mg twice daily >12 years.  Dose reduction:  if azole treatment. | aGvHD:  156 (range 78-969) after HSCT  cGvHD:  407 (range 127-1376) after HSCT | aGVHD:  immune suppressive agents before ruxolitinib: 4 (median)  cGVHD:  immune suppressive agents before ruxolitinib: 3.5 (median) | aGVHD:  Continuation for median 86 (range 35-308) days  cGVHD:  Continuation for median 126 (range 14-616) days | aGVHD:  Discontinued for adverse events (n=2) and death (n=2)  cGVHD:  Discontinued for adverse events (n=3) and death (n=1) |
| Escamilla Gomez, V. [16], 11 | Initial dose:  Median dose 10 mg twice daily | Not reported | aGVHD:  immune suppressive agents before ruxolitinib: 3 (range 1-5)  cGVHD:  immune suppressive agents before ruxolitinib: 3 (range 1-10) | Not reported | Discontinued for adverse events (n=3) and death (n=18) |
| Laisne, L. [14], 29 | Initial dose:  Median initial dose  <6 years 12.6 mg/m2/day  >6 years 12.8 mg/m2/day  Dose reduction:  No adjustment of the dose of ruxolitinib was performed in case of coadministration of azoles. | 91 days (range 17- 518) after HSCT | aGVHD and cGVHD:  immune suppressive agents before ruxolitinib: 2 (range, 1-6) | Continuation for median 183 (range 43-236) days | Discontinued for or CR (n=17) |
| Meng, G. [21], 3 | Initial dose:  5-10 mg twice daily (no other specification) | Not reported | cyclosporine, mycophenolate mofetil, and FK506 | Continuation for median 63 (range 24-206) day | Not reported |
| ﻿Moiseev, I. S. [13], 34 | Initial dose:  <40 kg: 0.15 mg/kg twice daily  >40 kg: 10 mg twice daily  Dose escalation:  increasing until a maximum of 10 mg twice daily | aGvHD:  16 (range 5-113) days after diagnosis  cGvHD:  376 (range 28-3219) days after diagnosis | aGVHD:  immune suppressive agents before ruxolitinib: 1 (range 1-2)  cGVHD:  immune suppressive agents before ruxolitinib: 2 (range 1-5) | aGVHD:  Continuation for median 5 months  cGVHD:  Continuation for median 23 months | Not reported |
| ﻿Schoettler, M. [24], 4 | Initial dose:  5 mg twice daily (all patients >40 kg)  Dose escalation:  10 mg twice daily  Dose reduction:  In one patient with severe renal insufficiency (2.5 mg 3 days/week) | Not reported | aGVHD and cGVHD:  immune suppressive agents before ruxolitinib: range, 3-6 | Continuation for median 7 months (range 2 months-1 year) | Discontinued for adverse event (n=1) |
| ﻿Uygun, V. [22], 29 | Initial dose:  <15 kg: 2.5 mg twice daily >15 kg: 5 mg twice daily  Dose escalation:  increasing until a maximum of 10 mg twice daily  Dose reduction:  if azole treatment. | aGvHD:  28 (range 7-231) days after diagnosis  cGvHD:  28 (range 7-52) days after diagnosis | aGVHD and cGVHD:  immune suppressive agents before ruxolitinib: range, 1-3 | aGVHD:  Continuation for median 3.6 (range 1.0-10.6) months  cGVHD:  Continuation for median 3.3 (range 2.0-11.3) months | Discontinued for death (n=3) |

**Supplementary Table S2.** Most common adverse events after ruxolitinib treatment in pediatric patients among the included studies

| **Author,**  **yea** | **No. of pts** | **Neutropenia – no. (%)** | **Thrombocytopenia – no. (%)** | **Liver impairment – no. (%)** | **Bacterial infections – no. (%)** | **Viral infections – no. (%)** | **Fungal infections – no. (%)** | **Other reported complications** | **Discontinuation for adv effects** |
| --- | --- | --- | --- | --- | --- | --- | --- | --- | --- |
| Khandelwal, P. ^38^ | 13 | 5 (46) | 3 (23) | 7 (54) | 6 (46) | 7 (54) | 1 (8) | 0 | 4 (30) |
| González Vicent, M. ^39^ | 22 | 0 | 1 (5) | 0 | 3 (14) | 8 (36) | 2 (9) | 0 | Not reported |
| ﻿Schoettler, M. ^45^ | 4 | 0 (0) | 0 (0) | 0 | 0 | 0 | 0 | 0 | 0 |
| ﻿Uygun, V. ^40^ | 29 | 7 (24) | 7 (24) | 2 (7) | Not reported | 6 (21) | Not reported | Myalgia-spasm (1) | Not reported |
| Laisne, L. ^41^ | 29 | 0 | 3 (10) | 0 | 0 | 12 (41) | Not reported | Not reported | 0 |
| Meng, G. ^46^ | 3 | 3 (100) | 3 (100) | 0 | 0 | 0 | 0 | Not reported | Not reported |
| Mozo, Y. ^37^ | 19 | 5 (26) | 5 (26) | 10 (52) | 7 (37) | 6 (32) | 2 (11) | Anemia (1) | 4 (21) |
| Yang, W. ^42^ | 53 | 16 (28) | 16 (28) | 8 (14) | 6 (5) | 11 (19) | 1 (2) | Not reported | 7 (13) |
| Marcuzzi, A. ^43^ | 12 | 2 (17) | 2 (17) | 0 (0) | Not reported | 9 (75) | Not reported | ﻿Dyslipidemia | Not reported |
| Wang, Y. ^47^ | 16 | 6 (30) | 3 (15) | 2 (10) | 4 (20) | 3 (15) | Not reported | Not reported | Not reported |
| Total (weitghted average) |  | 26% | 20% | 14% | 11% | 30% | 5% | - |  |

**Supplementary Table S3.** Summary of survival analysis among the included papers

| Author,  yea | No. of pts | % of patients surviving | OS % | EFS/DFS % | NRM % | Median time of follow up, Days (Range) | Causes of Death | Other analysis |
| --- | --- | --- | --- | --- | --- | --- | --- | --- |
| Khandelwal, P. [15] | 13 | 53,9 | / | / | / | 401 (219-969) from HSCT | 5 of 6 deaths attributable to GvHD (3 for aGvHD and 2 for bronchiolitis obliterans) | / |
| González Vicent, M. [19] | 22 | / | All cohort: 6m 62  aGvHD: 6m 30  cGvHD: 6m 100 | 58 | 28  (42 for severe aGvHD) | 716 (131-4775) from HSCT | 3 for aGvHD and 3 for viral infections | / |
| ﻿Schoettler, M. [24] | 5 | 100 | / | / | / | 730 (304–973) from HSCT | / | / |
| ﻿Uygun, V. [22] | 29 | 89,7 | / | / | / | / | 2 for infection and 1 for GvHD progression | / |
| Laisne, L. [14] | 29 | 79,3 | / | / | / | 85 (177-1042) from HSCT and 480 (93-829) from ruxolitinib start | 2 for infections (Adenovirus and Pseudomonas Aeruginosa) and 2 for worsening of GvHD | Rate of ORR significantly associated with survival |
| Meng, G. [21] | 12 | 66,7 | / | / | / | / | 2 for severe acute GvHD and 2 for severe infection | The median survival after HSCT was 64.6 weeks in the ruxolitinib group |
| ﻿Moiseev, I. S. [13] | 75 (34 pediatrics) | / | aGvHD: 59  cGvHD: 85 | aGvHD: 56  cGvHD: 74 | aGvHD: 34  cGvHD: 6 | 852 (700-1430) from ruxolitinib start | / | Worse outcome for aGvHD remained significant when corrected for underlying disease risk and response to ruxolitinib  No difference in OS between adults and children (65% vs 53%, p = 0.44) in both aGvHD and cGvHD.  The major predictor of survival in aGVHD group was grade III–IV gastrointestinal involvement, while for cGvHD only the underlying disease risk resulted significant |
| Mozo, Y. [12] | 19 | / | All cohort: 2y 71.9  aGvHD: 1y 64.8  cGvHD: 2y 76.4 | / | / | / | 3 of 4 deaths correlated to GvHD and infections | / |
| Yang, W. [20] | 53 | / | aGvHD: 6m 92,3  cGvHD: 6m 100 | aGvHD: 6m 69,3  cGvHD: 6m 96,9 | / | / | 4 aGvHD patients died for GvHD complications | / |
| Wang, Y. [17] | 20 | 90% | / | / | / | 652 (84–1127) from ruxolitinib start and 1331 (242–3450) from cGVHD diagnosis | 2 for GvHD progression |  |

**Supplementary Table S4.** Adverse events reported in <12 years children included in the single patient analysis

|  | **N** | **Cytopenia** | **Liver toxicity** | **Infections (all)** | **CMV reactivation** | **No adverse event** |
| --- | --- | --- | --- | --- | --- | --- |
| **All patients** | 80 | 16 (20%) | 16 (20%) | 16 (20%) | 8 (10%) | 42 (53%) |
| **aGvHD** | 26 | 7 (26%) | 10 (37%) | 6 (22%) | 4 (15%) | 7 (26%) |
| **cGvHD** | 24 | 4 (17%) | 6 (25%) | 10 (42%) | 4 (17%) | 14 (58%) |
